# Routine Frozen Section Analysis May Be Omittable for Nodular Basal Cell Carcinoma Undergoing Local Flap Reconstruction

**DOI:** 10.3390/cancers18152372

**Published:** 2026-07-23

**Authors:** Mads Koefoed Hansen, Mikail Topal, Iselin Saltvig, Taiba Alrasheed, Frederik Penzien Wainer Mamsen, Nicco Krezdorn

**Affiliations:** 1 Department of Plastic Surgery and Breast Surgery, Zealand University Hospital, SUH, 4000 Roskilde, Denmark; 2 Department of Orthopedic Surgery, Holbaek Hospital, 4300 Holbaek, Denmark

**Keywords:** non-melanoma skin cancer, frozen section analysis, local flap reconstruction, nodular basal cell carcinoma

## Abstract

**Background:** Intraoperative frozen section analysis (FSA) is commonly used to verify conclusive margins after resection of non-melanoma skin cancer (NMSC) before reconstruction. However, its necessity in nodular basal cell carcinoma (nBCC) remains inconclusive following local flap reconstruction. **Methods:** Retrospectively, this quality assessment study investigated consecutive patients undergoing FSA-guided excision of nBCC following local flap reconstruction. The study evaluates predictors for free margins after the first FSA. **Results:** Between 1 January 2024 and 20 January 2025, 112 patients underwent removal of biopsy-verified nBCC followed by local flap reconstruction. Ninety-two patients (82.1%) had conclusive margins on the first FSA with excision margins of 3–5 mm. Of 20 FSA-positive results, 12 were confirmed as true positives on paraffin embedding while eight were false positives, giving FSA a positive predictive value of 60% (12/20); FSA missed residual tumor in four patients (3.6%), corresponding to a sensitivity of 75% and a specificity of 91.7%. Referral biopsy type (punch or curettage) did not significantly affect the accuracy in predicting the BCC subtype, and no demographic, tumor, or location variable was independently associated with margin status. **Conclusions:** In this study, FSA led to further resection in 20 of 112 cases; however, with a positive predictive value of 60%, it only prevented a second surgery in 10.7% of cases. Because the present analysis is retrospective and lacks oncological follow-up, our findings should be regarded as hypothesis-generating rather than confirmatory: selective omission of routine FSA in well-defined nBCC may shorten the operative time and simplify the workflow but require prospective validation.

## Simple Summary

Basal cell carcinoma is the most common form of skin cancer and often appears on the face, where complete removal while preserving healthy tissue is important. Frozen section analysis can be performed during surgery to assess the edges of the removed tissue immediately so that any remaining tumor can be removed in the same operation. However, frozen section analysis adds time and cost and is not always reliable. In this study, we reviewed 112 patients who had a common type of basal cell carcinoma in the facial region. The tumor was removed and reconstructed with a local skin flap. We examined how often frozen section analysis could affect the surgery and how accurate it was. Our findings suggest that for selected tumors, this additional procedure may not always be necessary, although larger prospective studies are needed to confirm this.

## 1. Introduction

Basal cell carcinoma (BCC) is the most common cause of cancer in the Danish population, with more than 20.000 new patients diagnosed with BCC in Denmark annually [1] and with rising incidence globally [2]. BCC is classified as a non-melanoma skin cancer (NMSC), a term encompassing all non-melanoma malignancies of the skin arising from cells in the epidermis or dermis. BCC accounts for approximately 86% of all NMSC cases, while squamous cell carcinoma (SCC) constitutes around 14% in European populations [3]. The remaining types of NMSC comprise less than 1% of the total NMSC [4].

Five main clinical variants of BCC are widely recognized: nodular, superficial spreading, morphea/sclerosing, infiltrative, and pigmented [4]. Nodular BCC (nBCC) is the most common clinical variant and, together with the superficial spreading BCC, is considered a “low risk” variant, associated with the best prognosis in terms of cure rates and complications [2].

BCCs are predominantly located in the face, especially on the nose and the periocular region, rendering radical treatment of these tumors technically and cosmetically challenging [3,4,5,6], often requiring local flap reconstruction. Inconclusive tumor resection can have negative implications, as re-excision of the non-radical tumor border after primary resection can be even more challenging [7].

Treatments for NMSC are broadly categorized as surgical and non-surgical [8], with the gold standard being surgical intervention for nBCC at the surgeon’s discretion [9,10]. Removal of NMSC located in areas where excision could cause significant functional or cosmetic impairment is referred to as critically located. The patients may benefit from a narrower excision margin in such areas, e.g., perioral-, periorbital- and nasal regions, before reconstruction. To increase the chance of radical tumor removal, intraoperative frozen section analysis (FSA) or Mohs micrographic surgery (MMS) can be utilized [9].

MMS involves staged excision and examines 100% of the peripheral and deep margin of each stage in horizontal sections, allowing precise mapping of residual tumor and targeted re-excision only where necessary [11,12,13].

FSA-controlled excision is based on standard excision, in which resection margins are submitted for intraoperative frozen-section histology, and the surgeon awaits the pathologist’s report before wound closure or further resection [14].

This study aimed to investigate predictors for conclusive margins after the first FSA of biopsy-verified referred nBCC. Identification of such predictive factors may generate the hypothesis that selected subgroups of patients could be managed with standard excision alone. Because the present study includes no recurrence or follow-up data, this is presented only as a hypothesis for prospective testing rather than as a demonstrated safety conclusion; any such strategy would require validation in prospective studies with oncological follow-up before recurrence risk could be considered unaffected. This could prompt a re-evaluation of current practices for FSA for nBCC, optimizing patient care with respect to treatment efficacy, cost-effectiveness, and waiting times.

## 2. Materials and Methods

**Figure 1:**
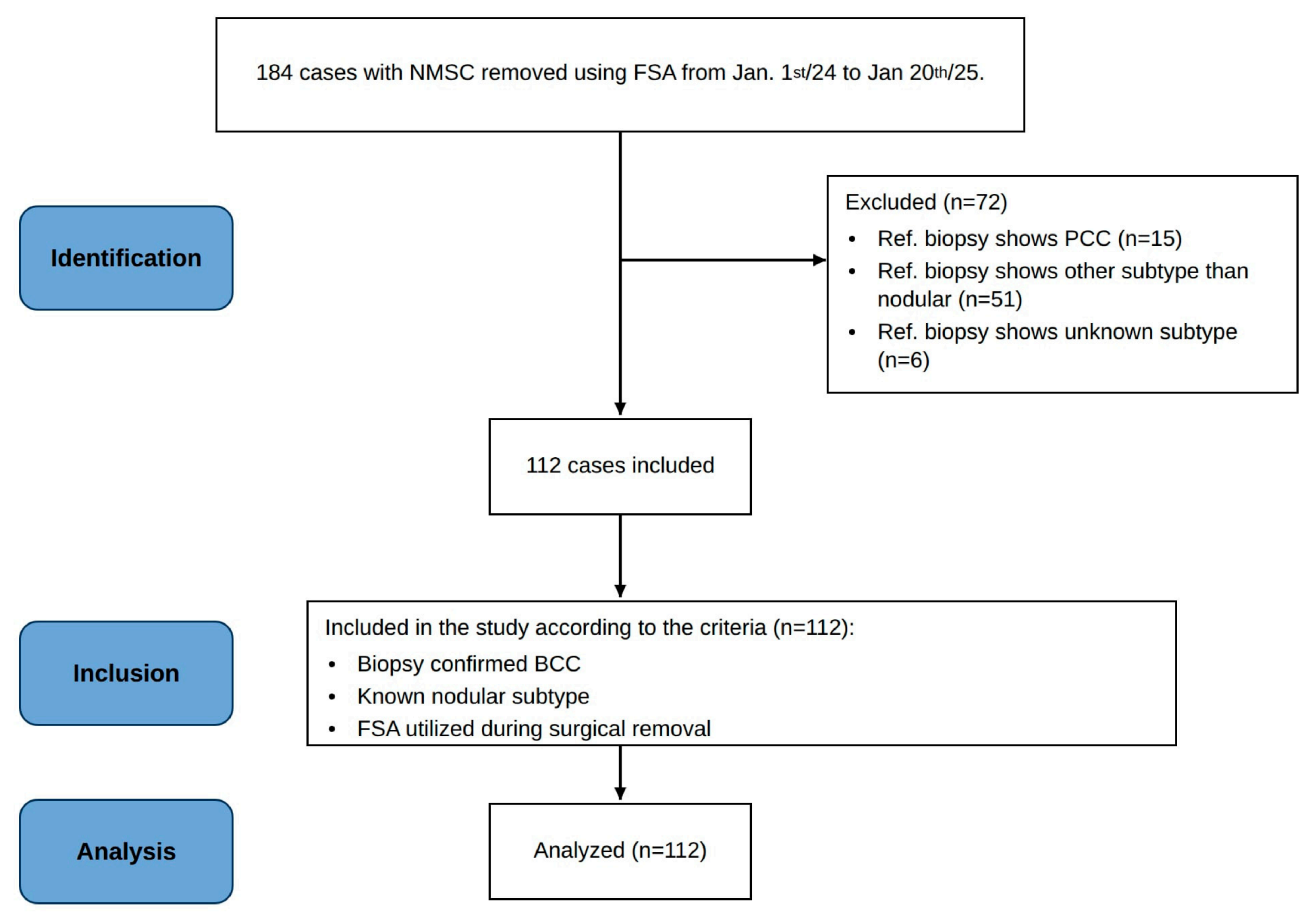
Flow diagram of patient inclusion.

This study is approved by the local hospital committee as a retrospective quality assurance study with the approval number EMN-2025-00153, and thus, no ethical committee approval was necessitated. The study complies with the General Data Protection Regulation (GDPR). Consecutively, patients referred to the Department of Plastic Surgery at Zealand University Hospital, Roskilde, Denmark, from 1 January 2024 to 20 January 2025 with biopsy- or curettage-verified nBCC and after preconsultation were operated on using an FSA guide, with a following local flap reconstruction. Data was collected from electronic medical records in accordance with local guidelines between October 2025 and December 2025. Patient data was obtained from the public healthcare system Sundhedsplatformen by EPIC using research and quality assurance templates, and approved data was transferred manually to an Excel file located in a secure hospital drive prefabricated for this study. The data was obtained, extracted, and analyzed by the first and last author independently. Questionable cases were reviewed by both data collectors. The study database comprised operations utilizing FSA, totaling 184 operations, of which 112 were nBCCs upon referral and are included in this study (Figure 1). Intraoperatively, the main specimen was excised along with 5 smaller biopsies from the margins. The main specimen was fixed in formalin and processed for paraffin embedding with hematoxylin and eosin (H&E) staining, and the 5 specimens for FSA consisted of four excised biopsies from the radial margins (corresponding to 12 o’clock, 3 o’clock, 6 o’clock, 9 o’clock) and one from the deep margins. All five margin samples were sent for FSA, and a verbal confirmation would go to the surgeon if the margins were clear or further resection was needed. Potential further excision would be performed, and it was at the surgeon’s discretion whether FSA was needed again before a following reconstruction. Postoperatively, all samples would be sent for paraffin-embedding histology as a control measure for the FSA.

FSA-guided procedures were performed by supervised junior residents, unsupervised senior residents, or consultants.

Data was analyzed using univariate and multivariate logistic regression analyses to assess the correlations between categorical and continuous predictors and the categorical outcome. Variables were included in the multivariate model based on clinical relevance. Results are presented as odds ratios (ORs) with 95% confidence intervals (CIs). A *p* value of 0.05 or less was considered statistically significant. The study is reported in accordance with the STARD 2015 statement for diagnostic accuracy studies [15].

## 3. Results

### 3.1. Patient Demographics

The study cohort included 112 patients with a mean age of 74.0 years (SD 10.2) and comprised 46 females (41.1%) and 66 males (58.9%) (Table 1). The majority were non-smokers (80.2%), 17.9% were active smokers, and 1.9% had paused smoking for 6 weeks before surgery. Regarding alcohol intake, one third reported no consumption (32.5%), just over half consumed 1–7 units per week (51.8%), and a minority reported more than 7 units per week (8.8%). Alcohol use was not reported in 5.3% of patients (Table 1).

### 3.2. Referral Data

Patients were referred to the Department of Plastic Surgery, Zealand University Hospital, Denmark, with different types of diagnostic biopsies suitable for histopathological confirmation and tumor subtype evaluation, most commonly punch biopsies of varying diameters (Table 2). Two-millimeter punch biopsies were the most common type, used in 60.4% of cases, followed by 3 mm punch biopsies and curettage biopsies, each accounting for 14.4% of cases. Less frequently, 4 mm punch biopsies and primary excision biopsies were used (5.4% each), and in 0.9% of patients, the initial biopsy type was not reported (Table 2).

### 3.3. Tumor Location, Size and Distribution

The tumor characteristics included a mean size of 8.9 mm (SD 4.0 mm), with a recurrence rate of 12.5% (Table 3). Tumors eligible for FSA were predominantly located in the nasal region (91.1%), whereas extra-nasal lesions on the frontal, periorbital, temporal, buccal, perioral and mental regions each accounted for ≤3% of cases (Table 3). Within the nasal region, lesions were most often situated on the nasal ala (58.0%) and apex nasi (21.4%), with fewer on the lateral nasal region (6.2%) and dorsum nasi (5.4%), and none on the soft triangle (Table 3).

During operative tumor excision, intraoperatively reported margins were 3 mm in 88.4% of patients, whereas 4 mm and 5 mm margins were used in 0.9% and 10.7% of patients, respectively, resulting in a pooled mean defect size of 15.4 mm (SD 4.4 mm) (Table 3).

### 3.4. Conclusive Margin Assessment

FSA revealed inconclusive margins in 20 of 112 resections (17.9%) (Table 4). Fifteen (13.4%) of these involved the lateral margins, three (2.7%) involved the deep margins, and two (1.8%) had simultaneous involvement of both lateral and deep margins.

After FSA, sections were embedded in paraffin for final histological analysis. FSA and paraffin sections were concordant in 100 patients (89.3%). While conclusive in FSA, four cases (3.6%) showed residual tumor cells in paraffin sections, and thus, were classified as “false clear FSA”. Eight cases (7.1%) showed no residual tumor cells in paraffin sections, while inconclusive in FSA, and thus, were classified as “false inconclusive FSA”.

When comparing it with paraffin embedding, FSA demonstrated 12 true inconclusive margins and four false conclusive margins, which leaves FSA with a sensitivity of 75%. And with eight false inconclusive and 88 true conclusive, this leaves FSA with a specificity of 91.7%. For post-test probability measures, the positive predictive value (PPV) is 60% (12/20), the negative predictive value (NPV) is 95.7% (88/92), and there is a complementary 40% false discovery rate (FDR).

Furthermore, comparison of preoperative biopsy results with final paraffin sections from the complete excision reported a discrepancy between basal cell carcinoma subtypes in 21 patients (18.8%) and complete absence of malignancy in seven patients (6.3%) (Table 4).

### 3.5. Predictors for Conclusive FSA Margins

Predictors for conclusive margins following the first FSA were evaluated using a univariate logistic analysis (Table 5) and a multivariate logistic regression analysis (Table 6). The single significant predictor for conclusive margins was narrower surgical margins (*p* = 0.006). No other demographic, tumor, or biopsy variables reached statistical significance in predicting conclusive margins. Recurrent tumors have a higher odds ratio (OR 2.05; *p* = 0.271) for conclusive margins.

### 3.6. Biopsy Type Predictive Value in Subtyping nBCC

Discrepancies in subtyping between the biopsy and the paraffin embedding of the final excision biopsy were observed in the beforementioned 21 cases (18.8%) (Table 7).

## 4. Discussion

This study reports utilizing FSA for the removal of 112 nBCCs and resulted in 17.9% (20 of 112) inconclusive margins needing further resection, which is lower than Nizamoglu et al. and Moncrieff et al. reporting 28% and 28.7%, respectively [16,17]. Nizamoglu et al. also reported 3–5 mm intraoperative margins, like our study [18,19], but this was not specified by Moncrieff et al. Our study reports that four of the 20 (20%) FSAs were falsely conclusive, resulting in additional re-excision after paraffin embedding. This is contradictory to Moncrieff et al. reporting only four out of 37 (9.7%) false negative FSAs [17].

The subset of false inconclusive margins following FSA was 7.1% (8 of 112) after paraffin-embedded validation, leading to additional removal of healthy tissue primarily in highly aesthetic facial regions. Current literature describes that FSA has a histologic accuracy between 83.3% [20] and 97.7% [21] compared with paraffin-embedded sections, which is why subsequent histopathological assessment of paraffin-fixed sections may reveal tumor cells in the margins. FSA has been reported to contribute to a false inconclusive rate of 2.0% (Otsuka et al.), which is considerably lower than reported in this study [22] (Table 4).

Intraoperative FSA, although generally accurate, may both under- and overestimate marginal status in a minority of cases, in line with previously reported discordance rates between frozen and paraffin sections in surgical pathology. Additionally, comparison of preoperative biopsy reports with paraffin sections from the definitive excision showed a different basal cell carcinoma subtype in 21 patients (18.8%) and complete absence of malignancy in seven patients (6.3%), reflecting the known limitations of diagnostic biopsies in reliably characterizing the histologic subtyping of basal cell carcinoma.

This study did not find a significant correlation between the type and size of samples performed upon referral (puncture biopsy, curettage or others), with an increased risk of finding a more invasive or other BCC subtype. This corresponds to an overall biopsy-to-paraffin concordance of 74.9% across all 112 cases, counting both the 21 cases with a different subtype (18.8%) and the seven cases with no malignancy on the definitive specimen (6.3%) as discordant. Previous evidence reports between 72% [23] and 82% [24] accuracy in BCC subtype, which is similar to the findings in this study. In this cohort, biopsy type was not significantly associated with correct subtyping (Table 7), and the small number of discordant cases limited any formal assessment of whether particular biopsy types or tumor characteristics predict upstaging to a more aggressive subtype. Patients should nonetheless be counselled preoperatively that the definitive subtype, and occasionally the presence of a residual tumor, may differ from the referral biopsy, irrespective of the biopsy technique used.

Surprisingly, the data report a 2.42 OR (*p* = 0.006) favoring a narrow excision margin. This has been discussed between authors, and we hypothesize that the surgeons decide to initially excise with wider margins when the tumor is not well-circumscribed. This has, to our knowledge, not been reported before and may be confounded by predictors that have not been collected in this dataset. Furthermore, as we mainly perform FSA on “critical areas,” this could represent a selection bias, as the data is only collected when FSA was performed. Because this association did not persist as an independent predictor and most plausibly reflects confounding by indication, the excision margin should not be interpreted as a causal determinant of margin status, and dedicated prospective studies would be required to evaluate it.

A predictor that is included in the decision determining the conclusive tumor margins is recurrence. However, this study did not find recurrent tumor as a relevant predictor for conclusive margins (OR 2.05; *p* = 0.271). Contrary to this, current literature supports increasing excision margins of recurrent tumors [25,26]. The result of our study may reflect a limited cohort size, or the previous tumor was not located in proximity to a previously excised area.

The value of FSA is clear; however, the cost/effectiveness could be a subject for discussion and would require larger cohort studies to clarify the risks and benefits. The increased risk of inconclusive margins when excisions are performed without FSA must be weighed against the possible challenges of reorientating the inconclusive margins following local flap reconstruction. Zingaretti et al. have proposed a list of methods to increase the safety of precision re-excision of inconclusive margins by establishing three rudimentary guidelines based on clear margin assessment and clinical experience [27].

Omitting routine FSA during NMSC removals could improve operational efficiency and reduce costs significantly. Currently, at this study site, an operating theater accommodates two NMSC cases with FSA and one minor case daily, utilizing 120 min per NMSC case (60 min for FSA) on average.

By eliminating FSA, the theater could increase its capacity by 1–2 NMSC removals daily, thus reducing patient wait times and surgical durations.

Even though this study did not find any patient demographic or tumor-characteristic predictors for minimizing the risk of conclusive margins, we believe an individualized approach must be considered when planning patients referred with NMSC in a functional- and cosmetically critical area. Further patient expectations and wishes must be considered before planning nBCC with or without FSA, especially in elderly patients with slow-growing, low-grade tumors such as nBCC [28]. For the purposes of this study, “selected”, “simple”, or “well-defined” nBCC refers operationally to a primary, non-recurrent, biopsy-verified nBCC that is clinically well-circumscribed and of the low-risk nodular subtype. Because the present cohort was surgeon-selected for FSA and heavily enriched for nasal and other critical-site tumors (91.1% nasal), we believe that any potential omission of FSA should be restricted to this narrowly defined group and should not be extrapolated to high-risk or aggressive subtypes, recurrent or poorly defined tumors, or anatomical sites underrepresented in this cohort.

The two types of FSA error carry different clinical consequences and should be weighed separately. A false-conclusive result—in which FSA reports a clear margin that is subsequently found to be involved on paraffin sections—occurred in four of 112 cases (3.6%) and is the more serious error, as it may leave a residual tumor. A false-inconclusive result—in which FSA prompts further resection that paraffin later shows to have been unnecessary—occurred in eight of 112 cases (7.1%) and primarily results in additional, potentially avoidable tissue removal. Any decision to omit routine FSA must therefore weigh the modest positive predictive value of 60% against this low, but not negligible, rate of false-conclusive results. Although case-level pathological re-review was not available in this retrospective dataset, several tissue- and processing-related factors may plausibly contribute to these discordant results. False-conclusive results may arise from sampling mismatch between the FSA and the definitive paraffin block, tangential or incomplete sectioning, or freezing and cautery artefact obscuring the tumor at the margin [29], as well as difficulty interpreting infiltrative strands on frozen material. False-inconclusive results may reflect freezing artefact, folded or overly thick sections, or over-interpretation of displaced benign structures. Prospectively recording these variables would help to separate true diagnostic limitations from correctable technical factors and to target quality improvement [30].

This study has several limitations. It is a retrospective, single-center quality-assurance analysis, and FSA was performed at the operating surgeon’s discretion rather than according to a fixed protocol; as a result, 91.1% of the included tumors were located on the nose, which limits the generalizability of the findings to other anatomical sites. The design includes no concurrent control group of nBCC excised without FSA, and no oncological follow-up or recurrence data were available; therefore, the analysis cannot directly demonstrate long-term oncological safety when compared with the current literature [31]. Finally, the number of inconclusive-margin events was small relative to the number of candidate predictors; the multivariable model should therefore be regarded as exploratory, and the non-converging estimates for some categories (odds ratios approaching zero with undefined confidence intervals) reflect data sparsity and complete separation rather than true effects. Future work could draw on larger, multi-center cohorts and may explore data-driven feature-selection methods to identify predictors of inconclusive margins. More specifically, prospective validation would ideally take the form of a multi-center study in which patients with operationally defined nBCC are allocated to FSA-guided versus standard excision using predefined inclusion criteria, standardized excision margins, and central histopathological review, with long-term oncological follow-up (for example, recurrence assessed at a minimum of five years) as the primary endpoint and a prespecified non-inferiority margin for recurrence. Combining such a design with penalized regression methods (for example, Firth’s correction) to accommodate sparse events would provide the reliable adjusted predictor estimates and safety data that the present retrospective analysis cannot.

## 5. Conclusions

This study found that further surgical resection prompted by positive FSA when removing nBCC only prevented a second surgery in 10.7% of the cases. This suggests that routine use of FSA in nBCC may be omittable in selected cases. Omission of FSA when removing simple nBCC could shorten the surgical time and simplify the operative workflow, improving patient care and waiting lists without compromising margin clearance in this cohort. However, because no oncological follow-up or recurrence data were available, prospective studies with long-term follow-up are required. Further patient expectations and wishes must be considered when planning tumor excision with or without FSA.

## Figures and Tables

**Table 1:** Patient demography.

Demographic		Total No. of Patients 112 (100%)
Gender (female)		46 (41.1%)
Age (years)		74.0 (SD = 10.2)
Smoker		
	None	85 (80.2%)
	Paused for 6 weeks	2 (1.9%)
	Active	19 (17.9%)
Alcohol		
	None	37 (32.5%)
	1–7 units/week	59 (51.8%)
	Above 7 units/week	10 (8.8%)
	Not reported	6 (5.3%)

**Table 2:** Referral biopsies.

Histological Assessment		Total No. of Patients 112 (100%)
Initial type of biopsy		
	2 mm punch biopsy	67 (60.4%)
	3 mm punch biopsy	16 (14.4%)
	4 mm punch biopsy	6 (5.4%)
	Curettage biopsy	16 (14.4%)
	Excision biopsy	6 (5.4%)
	Unreported	1 (0.9%)

**Table 3:** Tumor and surgical demographics.

nBCC Parameter			Total No. of Patients 112 (100%)
Size			8.9 mm (SD = 4.0 mm)
Recurrence			14 (12.5%)
Lesion location			
	Frontal region		1 (0.9%)
	Periorbital region		1 (0.9%)
	Temporal region		1 (0.9%)
	Buccal region		2 (1.8%)
	Perioral region		3 (2.7%)
	Mental region		2 (1.8%)
	Nasal region		102 (91.1%)
		Apex nasi	24 (21.4%)
		Ala nasi	65 (58.0%)
		Soft triangle	0 (0.0%)
		Dorsum nasi	6 (5.4%)
		Lateral nasal region	7 (6.2%)
Excision margin			
	3 mm		99 (88.4%)
	4 mm		1 (0.9%)
	5 mm		12 (10.7%)
Defect size			15.4 mm (SD = 4.4 mm)

**Table 4:** Conclusive margins following FSA.

Histological Assessment		Total No. of Patients 112 (100%)
Inconclusive frozen section analysis		20 (17.9%)
	Deep	3 (2.7%)
	Borders	15 (13.4%)
	Both	2 (1.8%)
Discrepancy between FSA and paraffin sections		
Paraffin confirms FSA		100 (89.3%)
	False conclusive FSA	4 (3.6%)
	False inconclusive FSA	8 (7.1%)
Discrepancy between biopsy results and paraffin embedding		Different BCC subtype: 21 (18.8%)
		No malignancy on definitive specimen: 7 (6.3%)

**Table 5:** Univariate logistic regression analysis for free margins after FSA.

Predictors for Clear FSA Margins			ODDS Ratio	95% CI	*p* Value
Age (years)			1.02	0.97–1.08	0.392
Gender (male)			1.79	0.66–5.45	0.271
Smoker (active)			0.47	0.07–1.86	0.343
Smoker (pause for 6 weeks)			0.00	NA–NA	0.993
Alcohol (below recommendations)			0.74	0.26–2.14	0.569
Alcohol (above recommendations)			0.40	0.02–2.66	0.420
Size			1.05	0.93–1.18	0.445
Excision margin			2.42	1.28–4.61	0.006 *
Lesion			1.09	0.98–1.21	0.127
Recurrence (yes)			2.05	0.51–7.01	0.271
Biopsy type (3 mm punch)			0.21	0.01–1.18	0.148
Biopsy type (4 mm punch)			0.64	0.03–4.35	0.691
Biopsy type (curettage)			0.46	0.07–1.87	0.331
Biopsy type (excision)			0.00		-
Baseline = nasal region	Frontal region		0.00		-
	Periorbital region		0.00		-
	Temporal region		0.00		-
	Buccal region		0.00		-
	Perioral region		0.00		-
	Mental region		0.00		-
	Baseline = other than nasal region	Apex nasi	0.61	0.12–3.62	0.568
		Ala nasi	0.42	0.10–2.22	0.266
		Dorsum nasi	1.17	0.11–10.46	0.889
		Lateral nasal region	0.00		-

* Smoker (pause for 6 weeks) presented with non-convergent estimates as a result of data sparsity, shown for the sake of accuracy.

**Table 6:** Exploratory (sensitivity) multivariate logistic regression analysis for conclusive margins after the first frozen section analysis. This model is presented as an exploratory sensitivity analysis only, given the small number of inconclusive-margin events and the resulting data sparsity (non-convergent estimates for several categories) and is not intended to support claims of independent prediction.

Predictors for Clear FSA Margins			ODDS Ratio	95% CI	*p* Value
Age (years)			1.00	0.94–1.07	0.961
Gender (male)			2.65	0.75–10.04	0.148
Smoker (active)			0.37	0.04–2.30	0.333
Alcohol (below recommendations)			0.58	0.14–2.37	0.448
Alcohol (above recommendations)			0.44	0.02–4.35	0.529
Size			0.99	0.84–1.15	0.867
Excision margin			2.42	1.01–6.28	0.051
Recurrence (yes)			1.68	0.23–10.03	0.271
Biopsy type (3 mm punch)			0.38	0.02–2.73	0.408
Biopsy type (4 mm punch)			0.72	0.03–6.44	0.794
Biopsy type (curettage)			0.35	0.03–2.20	0.314
Biopsy type (excision)			0.00	NA–NA	
S location	Baseline = other than nasal region	Apex nasi	0.51	0.05–5.57	0.565
		Ala nasi	0.52	0.07–4.70	0.534
		Dorsum nasi	0.94	0.05–10.64	0.966
		Lateral nasal region	0.00	NA–NA	-

* Smoker (pause for 6 weeks) was not included because of few observations. This did not affect the results of the other predictors.

**Table 7:** Predictive value of biopsy type for correct subtyping.

Biopsy Type: Curettage Biopsy	OR	95% CI	*p* Value
2 mm puncture biopsy	0.86	0.25–3.46	0.817
3 mm puncture biopsy	0.18	0.01–1.45	0.153
4 mm puncture biopsy	0.55	0.02–5.08	0.630
Excision biopsy	0.00	NA–NA	

## Data Availability

The data presented in this study are available on request from the corresponding author due to ethical and data-protection restrictions under the General Data Protection Regulation (GDPR) and the study’s quality assurance approval (EMN-2025-00153).
